# mACPpred: A Support Vector Machine-Based Meta-Predictor for Identification of Anticancer Peptides

**DOI:** 10.3390/ijms20081964

**Published:** 2019-04-22

**Authors:** Vinothini Boopathi, Sathiyamoorthy Subramaniyam, Adeel Malik, Gwang Lee, Balachandran Manavalan, Deok-Chun Yang

**Affiliations:** 1 Graduate School of Biotechnology, College of Life Science, Kyung Hee University, Yongin-si 17104, Gyeonggi-do, Korea; vinothini9327@gmail.com; 2 Research and Development Center, Insilicogen Inc., Yongin-si 16954, Gyeonggi-do, Korea; moorthy@insilicogen.com; 3 Department of Biotechnology, Dr. N.G.P. Arts and Science College, Coimbatore, Tamil Nadu 641048, India; 4 Department of Microbiology and Molecular Biology, College of Bioscience and Biotechnology, Chungnam National University, Daejeon 34134, Korea; adeel@procarb.org; 5 Department of Physiology, Ajou University School of Medicine, Suwon 443380, Korea

**Keywords:** anticancer peptides, support vector machine, feature selection, optimal features, sequential forward search

## Abstract

Anticancer peptides (ACPs) are promising therapeutic agents for targeting and killing cancer cells. The accurate prediction of ACPs from given peptide sequences remains as an open problem in the field of immunoinformatics. Recently, machine learning algorithms have emerged as a promising tool for helping experimental scientists predict ACPs. However, the performance of existing methods still needs to be improved. In this study, we present a novel approach for the accurate prediction of ACPs, which involves the following two steps: (i) We applied a two-step feature selection protocol on seven feature encodings that cover various aspects of sequence information (composition-based, physicochemical properties and profiles) and obtained their corresponding optimal feature-based models. The resultant predicted probabilities of ACPs were further utilized as feature vectors. (ii) The predicted probability feature vectors were in turn used as an input to support vector machine to develop the final prediction model called mACPpred. Cross-validation analysis showed that the proposed predictor performs significantly better than individual feature encodings. Furthermore, mACPpred significantly outperformed the existing methods compared in this study when objectively evaluated on an independent dataset.

## 1. Introduction

The complex process by which normal cells are transformed to abnormal cancer cells is known as carcinogenesis or tumorigenesis [1]. Such processes may be attributed to several factors, such as hereditation [2], environment [3], or a change in the physiological microenvironment of the affected cells [4]. Thus, most cancers—regardless of the driving factors—are distinguished by the gradual accumulation of genetic modifications in the founder cells [5]. Generally, the division and differentiation of normal cells are strictly regulated by several signaling pathways. However, sometimes normal cells escape these signals, leading to uncontrolled growth and proliferation, which ultimately leads to cancer [1]. According to the world health organization (WHO), the most common types of cancers are lung, liver, colorectal, stomach, prostate, skin and breast [https://www.who.int/news-room/fact-sheets/detail/cancer]. Due to cancer, millions of deaths have been reported each year from both developing and economically-advanced countries. In 2018, it was anticipated that about 18 million new cancer cases and over 9 million deaths could occur due to cancer [6], and these deaths could reach well over 13 million by 2030 [7]. In the United States (US) alone, approximately 1.7 million new cancer cases and over 600,000 cancer related deaths are estimated for 2019 [8].

Traditional methods for the treatment of cancer includes surgery, radiation therapy and chemotherapy, which may also depend on the location, stage of the disease, and the patient condition [9]. Despite advances, these methods are expensive and can often exhibit damaging effects on normal cells. Additionally, there is a growing concern that cancer cells may develop resistance to chemotherapy and molecularly-targeted therapies [10]. Moreover, cancer cells are known to develop multidrug resistance through a broad range of mechanisms, which not only makes these cells resistant to the drug in use for treatment, but also several other compounds [11]. As soon as the molecular mechanism behind cancer (or, as a matter of fact, any disease) is understood, the next logical step is to discover a desirable remedy for it [12]. Therefore, in view of the above, there is an urgent need to discover and design novel anti-cancer drugs to combat this deadly disease.

During the last few decades, the role of peptides as anti-cancer therapeutic agents has been promising, which is apparent from various strategies available to address the advancement of tumor growth and spreading of the disease [13]. These anti-cancer peptides (ACPs) have shown the potential to inactivate various types of cancer cells [11]. ACPs are short peptides (typically 5–50 amino acids in length) that exhibit high specificity, high tumor penetration and ease of synthesis and modification, in addition to low cost of production [14,15]. In general, most of the ACPs exhibit either an α-helical or a β-sheet conformation, however, in some cases, extended structures have also been identified [16]. ACPs can be classified into two major groups; i) peptides that are toxic to both cancerous and normal cells, exhibiting little evidence of selectivity, and ii) peptides that are toxic to cancer cells, but not to normal mammalian cells and erythrocytes [11]. The mechanisms by which these ACPs affect cancer cells are not yet completely understood. However, the role of membranolytic or non-membranolytic mechanisms are implicated [11]. Furthermore, the mechanisms that are involved in the inhibition of certain biological processes, such as angiogenesis, protein–protein interactions, signal transduction pathways, and gene expression, including the inhibition of enzymes or proteins, have also been highlighted [13].

Since most of the ACPs are derived from protein sequences [17], the discovery of novel ACPs for cancer treatment will be a focus of research for future studies. It is expected that the number of ACPs will increase with the rapid growth of protein sequences in public databases as a consequence of high-throughput sequencing projects [15]. Identification and development of novel ACPs from experimental methods is expensive and extremely time consuming. Therefore, it is essential to develop sequence-based computational methods to rapidly identify potential ACP candidates from the sequencing data prior to their synthesis. In this study, we constructed a lowest redundancy benchmark dataset and used this for the development of a prediction model. To develop a prediction model, we explored seven feature encodings, including amino acid composition (AAC), dipeptide composition (DPC), composition-transition-distribution (CTD), quasi-sequence-order (QSO), amino acid index (AAIF), binary profile (NC5), and conjoint triad (CTF). To exclude irrelevant features on each of the feature encodings, we applied a two-step feature selection protocol and identified their corresponding optimal feature-based models. Finally, the predicted probability obtained from seven feature encoding models were used as an input to a support vector machine (SVM) to construct the final model called mACPpred. Furthermore, our proposed method (mACPpred) achieved consistent performance on both benchmark and independent datasets.

## 2. Results

### 2.1. Performance of Various Feature Encodings

**Figure 1:**
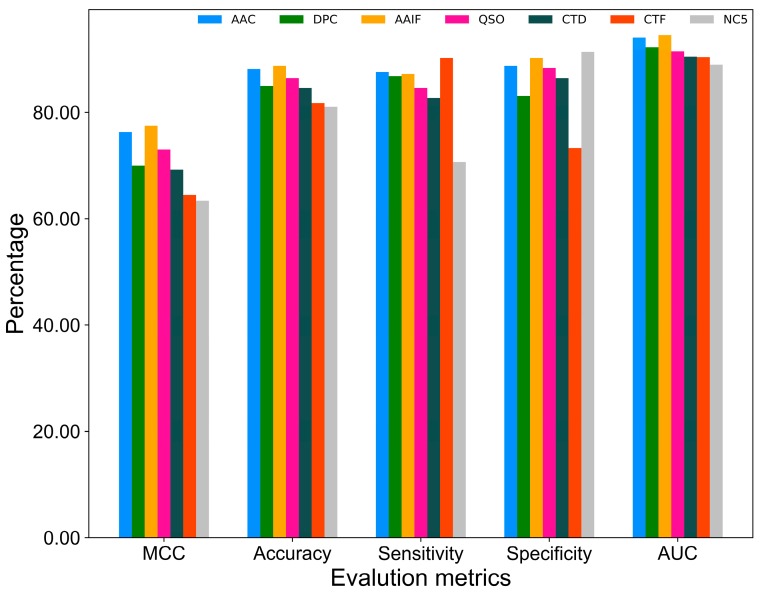
Performance of various feature encodings in a 10-fold cross-validation.

Firstly, we examined the capability of each feature encoding in classifying ACPs from non-ACPs. It should be noted that optimal machine-learning (ML) parameters for each feature encoding was obtained by conducting 10 independent 10-fold cross-validations. The best performance achieved by each feature encoding is shown in Figure 1. Results show that AAIF achieved the best performance with an accuracy of 88.72%, while AAC-, QSO-, DPC-, CTD-, CTF-, and NC5-based performance ranked at positions 2 to 7, respectively. Overall, seven feature encodings achieved a reasonable performance with an accuracy ranging between 81.0 and 88.7%. Furthermore, we observed that low-ranked feature encodings achieved the highest sensitivity and specificity. For instance, CTF achieved the highest sensitivity of 90.0%, which is 1.5–20% higher than the other encodings. Similarly, NC5 achieved the highest specificity of 91.35%, which is 1.06–18.0% higher than the other encodings. Although the basic nature of each feature encoding covers a different aspect of sequence information, each of these contribute towards better prediction. Therefore, it is essential to integrate these seven feature encoding-based models into a single model to overcome the limitations of each model and achieve a more balanced and stable performance.

### 2.2. Comparison of SVM and Other Classifiers

**Figure 2:**
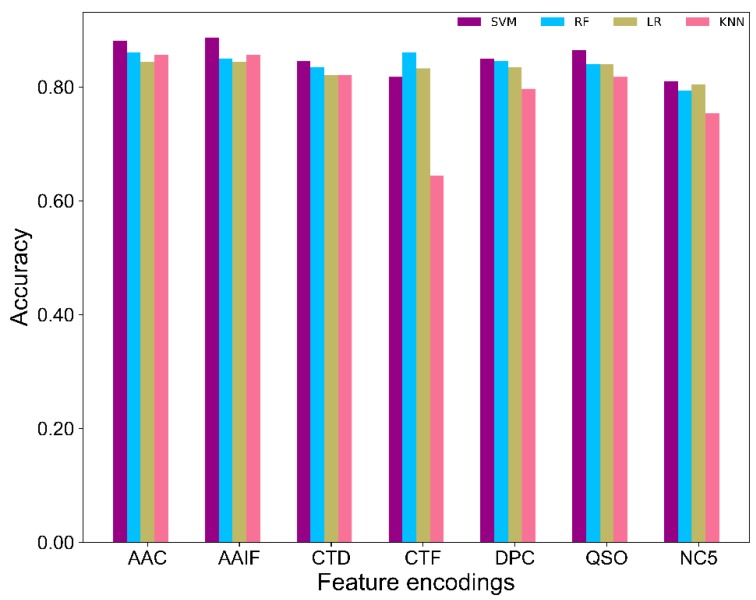
Comparison of SVM with other classifiers on seven different feature encodings.

To evaluate the effectiveness of SVM classifiers, we compared the performance of SVM-based classifiers against three other commonly used ML classifiers, namely random forest (RF), k-nearest neighbors (KNN) and logistic regression (LR), on seven feature encodings [18]. Using a 10-fold cross-validation test, the performance of the three other methods is shown in Table S1 and Figure 2. Results showed that SVM performed consistently better than the three other classifiers on six out of seven feature encodings. Precisely, the average accuracy achieved by SVM was ~1.1% higher than RF, ~2% higher than LR, and ~6% higher than KNN, indicating that SVM has a slight advantage over other methods in classifying ACPs from non-ACPs. Hence, we utilized only the SVM classifiers for further analysis.

### 2.3. Selection of the Optimal Features for Each Encoding

**Figure 3:**
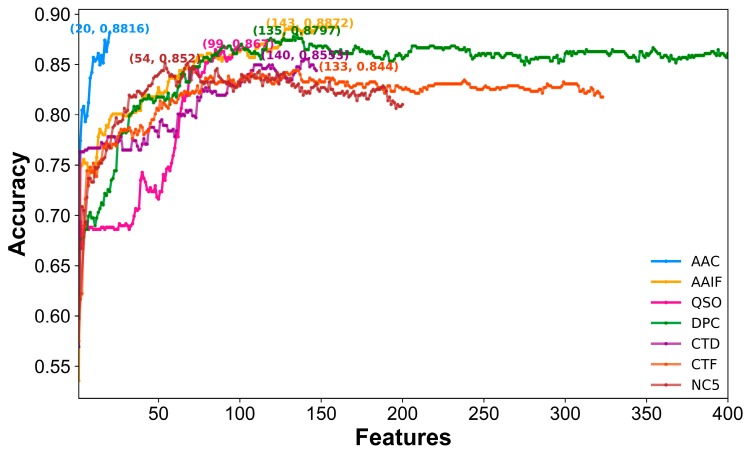
Sequential forward search for discriminating between anticancer peptides (ACPs) and non-ACPs. The maximum accuracy obtained from 10-fold cross-validation is shown for each feature encoding.

Since DPC, CTF and other encodings have a larger dimension, some of the features may be redundant or not all of them will be equally important. Therefore, it is mandatory to apply a feature selection protocol to remove redundant and irrelevant features. There are various feature selection techniques available in the literature [19,20,21,22,23], however, inspired by recent studies [24,25,26], we applied a two-step feature selection procedure to check whether it was able to reduce feature dimensions and improve performance. In particular, the F-score algorithm for ranking features (present in each feature encoding) was employed, followed by a sequential forward search to find the optimal feature set (Figure 3). Table 1 shows the number of features significantly reduced in the case of DPC (66.25%), CTF (58.82%), and NC5 (73%). On the other hand, a slight reduction can be observed in the case of AAIF (4.76%), QSO (1.0%), and CTD (10.62%). No reduction was witnessed in the case of AAC.

**Figure 4:**
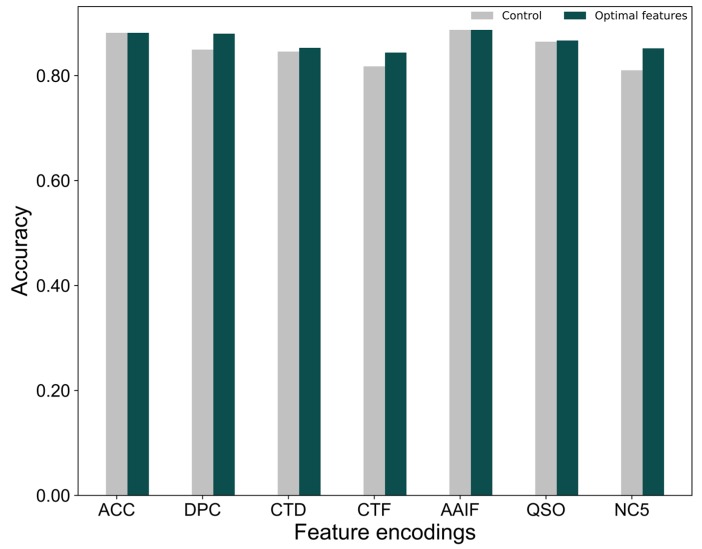
Performance comparison between the optimal feature set-based model against the respective controls (using all features).

Next, we examined the performance of each feature encoding based on their optimal features and compared this with the respective control (using all features). Figure 4 shows a significant improvement in the performance for three feature encodings, NC5, DPC and CTF, by 4.18%, 3.08% and 2.63% respectively, as compared to their control. CTD and QSO improvement is marginal (<1%), while no improvement is observed in AAC and AAIF. Although no improvement was observed in AAIF, the number of feature dimensions is slightly reduced. In the case of AAC, all the features are equally important for achieving the best performance.

To examine whether the optimal features are better than the excluded features for each feature encodings, we developed excluded features-based prediction models using the procedure as described in Section 2.1, and compared their performance with the control (using all features) and the optimal features. Notably, only four feature encodings (CTD, CTF, DPC, and NC5) were used for this analysis, while the remaining three feature encodings (AAC, AAIF, and QSO) were excluded considering the size of the optimal feature dimension and the control were similar. Figure S1 shows that the optimal feature-based models are consistently better than the control and also excluded feature-based models. Explicitly, the average accuracy achieved by the optimal feature-based models is 16.8% higher than the excluded feature-based models, and 2.7% higher than the control. This indicates that a two-step feature selection protocol selected more important features, thereby contributing to an improved performance. The optimal features for each feature encoding are provided in Table S2.

### 2.4. Construction of the Final Predictor

**Figure 5:**
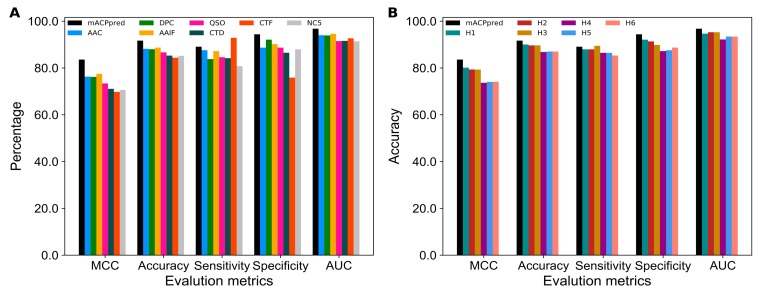
(**A**) Performance comparison of mACPpred with the single feature models, based on optimal features. (**B**) Performance comparison between mACPpred and hybrid features-based models.

The optimal feature-based model obtained for each feature encoding was used in the development of a final prediction model. Some of the previous methods used hybrid features (a linear combination of various feature encodings) as an input to a ML classifier for the development of a prediction model without any feature selection techniques [27]. However, we considered only the predicted probability of ACPs (values in the range of 0.0 to 1.0) from seven individual optimal models as input features to SVM and developed a final prediction model called mACPpred. Our proposed predictor achieves an Matthews correlation coefficient (MCC), accuracy, sensitivity, specificity and area under the curve (AUC) of 0.836, 0.917, 0.891, 0.944, and 0.968, respectively. To show the effectiveness of mACPpred, we compared its performance with seven feature encoding predictors (Figure 5A). Specifically, the MCC and accuracy of the proposed predictor was 4.6–13.8% and 3.5–7.3% higher than the individual predictors, thus indicating the effectiveness of our approach by integrating various feature encodings, which in turn contributes to an improved performance.

It might be possible that methods employing hybrid features (a combination of different feature encodings) perform better than the current approach because they utilize multiple elements and also complete the feature space. To investigate this possibility, we developed six hybrid-feature-based models using the following procedure: (i) Seven feature encodings were ranked according to the accuracy obtained from base-line models (Figure 1) and incorporated with AAIF one by one (H1: AAIF+AAC; H2: AAIF+AAC+QSO; H3: AAIF+AAC+QSO+DPC; H4: AAIF+AAC+QSO+DPC+CTD; H5: AAIF+AAC+QSO+DPC+CTD+CTF; H6: AAIF+AAC+QSO+DPC+CTD+CTF+NC5). Each of the hybrid features were used as an input to SVM and their corresponding models were developed using the same procedure as described in Section 2.1. Figure 5B shows the performance comparison of mACPpred with the hybrid-feature-based models, where mACPpred performed better with an MCC and accuracy value 3.46–9.5% and 1.7–4.7% higher than the hybrid models, respectively, thereby demonstrating the advantage of our approach in achieving the best performance.

### 2.5. Performance Comparison on the Independent Dataset

There are several examples where the prediction model showed an excellent performance during cross-validation. However, these performances are not transferrable while evaluating an independent dataset. Hence, an independent evaluation is needed to validate the robustness of the proposed method. Importantly, the independent dataset constructed in this study did not share greater than 90% sequence identity with our training dataset and other existing methods’ training datasets. We compared the performances of mACPpred with the previous methods, such as MLACP and iACP. It should be noted that MLACP contains two prediction models based on RF (RFACP) and SVM (SVMACP), and both the models were considered for comparison.

**Figure 6:**
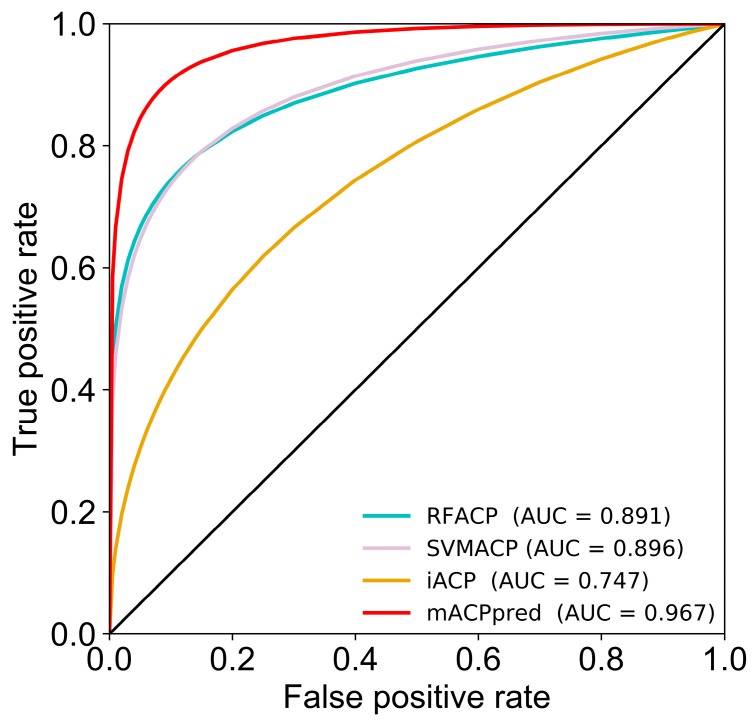
Comparison of binormal receiver operating characteristics (ROC) curves for ACPs prediction using different methods on independent dataset.

Table 2 shows that mACPpred achieves an MCC, accuracy, sensitivity, specificity, and AUC of 0.829, 0.914, 0.885, 0.943, and 0.967, respectively. More specifically, the MCC and accuracy of mACPpred is 23.7–49.1% and 14.6–33.4% higher, respectively, than the other methods compared in this study, demonstrating that the proposed method is capable of achieving an encouraging performance. Note that it is hard to get statistical estimation from the above threshold-based comparison. Hence, we utilized rank-based comparison using ROC [28], where two AUC values of different methods were assessed by a two-tailed test, from which the *p* value for the observed differences were obtained [29]. Table 2 and Figure 6 shows that the mACPpred significantly outperformed the existing predictors on the independent dataset.

### 2.6. Webserver Implementation

mACPpred webserver is freely accessible at the following link: www.thegleelab.org/mACPpred. Users can upload or paste query peptide sequences in the FASTA format, and after submitting peptide sequences, retrieve results in a separate interface. All datasets used in this study can be downloaded from the following link: http://thegleelab.org/mACPpred/ACPData.html, to check the reproducibility of our findings.

## 3. Discussion

In this study, we developed a novel predictor called mACPpred to predict ACPs from the given peptide sequence. To develop a predictor, a two-step feature selection protocol was applied on seven feature encodings (AAC, DPC, CTD, CTF, AAIF, QSO, and NC5) to obtain optimal feature-based prediction models, whose predicted probabilities of ACPs were further used as a feature vector. Finally, the probabilistic feature vector was used as an input to SVM for the development of a final prediction model. The benchmark and independent validation demonstrated that the mACPpred was able to clearly outperform existing predictors compared in this study for ACPs prediction. The novelty of our method is as follows: (i) The benchmark or training dataset has the lowest redundancy among the datasets reported in the literature; (ii) among various feature encodings employed in this study, this is the first instance where CTF and QSO are employed in ACP prediction, and (iii) most of the existing predictors either utilize single feature encodings or a combination of multiple feature encodings, hence, their feature dimension is very high. However, we have used only seven probabilistic features that cover a wide range of features (position specific, physicochemical, and compositional information). Basically, it transforms the complex high-dimensional feature into a low-dimensional one, further facilitating better discrimination between ACPs and non-ACPs.

Moreover, our approach can be applied to other sequence-based prediction problems, including post-translational modifications, peptide function predictions, and DNA/RNA function predictions. Although the proposed predictor has shown an excellent performance over the other methods, there is still room for improvement. This includes exploration of other ML algorithms such as decision tree-based [31,32] and neural network-based algorithms [33,34,35] on the same dataset, incorporation of novel features and computational approach as implemented in References [36,37,38,39], and increasing the size of the training dataset based on the future experimental data. Furthermore, we implemented our proposed algorithm in the form of user-friendly web-server (http://thegleelab.org/mACPpred) for the wider research community to use. We expect that mACPpred will be helpful for identifying novel potential ACPs.

## 4. Materials and Methods

### 4.1. Dataset Collection and Processing

We generated positive samples by utilizing the previously reported datasets of Tyagi et al. [40], Wei et al. [15], and Chen et al. [30], which contain 225, 250, and 288 (both training and independent datasets) experimentally verified ACPs, respectively. From this, we excluded sequences >50 amino acid residues because these may form outliers during prediction model development as very few peptides have larger than 50 amino acids. Subsequently, we applied a CD-HIT [41] threshold of 0.8 and excluded the redundant sequences, which resulted 266 positive samples. It should be noted that lower thresholds of the sequence identity (less than 50%) might reduce the sequence homology bias and could improve the model credibility. However, using higher threshold was necessary due to the smaller dataset size. For the collection of negative samples, we utilized 2250 samples reported by Tyagi et al. [40], wherein AMPs had been extracted from several databases including, APD, CAMP, DADP, for which no anticancer activity has been reported in the literature. Subsequently, we applied CD-HIT of 0.8 against the positive samples and among negative samples that resulted 2069 non-ACPs.

Training dataset: We generated a high-quality training dataset by selecting 266 ACPs and randomly selecting 266 non-ACPs from the 2069 non-ACPs set mentioned above. Generally, ML classifiers tend to produce unbiased performance on a balanced dataset [15], hence we selected an equal number of non-ACPs with ACPs. To the best of our knowledge, our training dataset has the lowest redundancy among the reported datasets, which was utilized to develop a prediction model.

Independent dataset: we manually collected positive samples (ACPs) from the following databases: DADP [42], DBAASP [43], DRAMP [44], and LAMP [45]. Subsequently, we applied a CD-HIT threshold of 0.9 among the collected peptides and also against the training dataset, which resulted in 157 ACPs. Furthermore, 157 non-ACPs were randomly selected from Tyagi’s negative dataset, mentioned above, which did not overlap with the training and independent positive datasets. This dataset was used to evaluate our prediction model. All the datasets utilized in this study can be found in the Supplementary Information.

### 4.2. Feature Extraction

To develop or train a prediction model, it is essential to formulate a diverse length of peptides as a fixed length of feature vectors. In this study, we explored seven different feature encodings that can be grouped into sequence-based features and physicochemical properties-based features, as described below:

#### 4.2.1. Sequence-Based Features

The differences between peptides can be reflected by amino acid sequences, which includes composition, profiles, physicochemical properties, permutation and combination modes of amino acids. Hence, we extracted five types of sequence-based features: AAC; DPC, QSO, CTF, and NC5.

1. AAC has been widely used in numerous sequence-based prediction tasks [46], which represents the occurrence frequencies of 20 standard amino acids in a given peptide sequence that generates a 20-dimensional vector.
2. DPC encoding of the given peptide sequence results in a fixed length of a 400-dimensional feature vector that summarizes amino acids fraction, the sequence-order, and fragment information.
3. QSO encoding of the given peptide sequence results in a fixed length of a 100-dimensional feature vector, by measuring the physicochemical distance between the amino acids within the sequence. A detailed description of QSO feature encoding along with a set of equations has been provided in previous studies [47,48,49].
4. CTF encoding generates a 343-dimensional feature vector for a given peptide sequence by clustering amino acids into seven classes according to their dipoles and side-chains volumes. A detailed description of CTF with a set of equation has been provided in previous studies [50,51,52].
5. In NC5, each amino acid is encoded as a 20-dimensional 0/1 vector. For example, the amino acid of type A and type C are encoded as (1,0,0,0,0,0,0,0,0,0,0,0,0,0,0,0,0,0,0,0) and (0,1,0,0,0,0,0,0,0,0,0,0,0,0,0,0,0,0,0,0), respectively. BPF(w) dimension is 20 × w, where w is sequence window length. Since the minimal length of the peptide in our dataset is 5, we fixed at this value and considered both N-and C- terminals (NC5) that generate a 200-dimensional vector.

#### 4.2.2. Physicochemical Properties-Based Features

Physicochemical properties have been widely used and successfully applied in numerous prediction problems including proteins [23,24,36], RNA [53,54], and DNA [38,55,56]. Here, we used the following two types: AAIF and CTD; both represent a global composition of amino acid properties in a different perspective.

1. In AAIF, we used only eight high-quality amino acid indices as reported in a previous study [57], which are LIFS790101 [58], CEDJ970104 [59], MIYS990104 [60], NAKH920108 [61], TSAJ990101 [62], MAXF760101 [63], BIOV880101 [64], BLAM930101 [65]. AAIF generates a 160 (=20 amino acids * 8 properties) dimensional vector, which has been successfully applied in numerous sequence-based prediction tasks [66,67,68].
2. We used seven different types of physicochemical properties listed in Table 3 where 20 standard amino acids are classified into 3 different classes according to their attributes. In CTD, composition, transition, and distribution are respectively encoded as a 21, 21, 105-dimensional feature vector. A detailed description of CTD with a set of equations has been provided in previous studies [69,70].

### 4.3. Support Vector Machine

SVM is one of the powerful machine learning algorithms, which has been widely used in numerous fields within bioinformatics [21,22,53,71,72,73]. The *scikit-learn* (v.0.19.1) library in Python was used to implement SVM algorithm [74]. The main objective of SVM is to find the optimal hyperplane that can maximize the distance between two categories (positive and negative) in high-dimensional feature space. We used the radial basis function (RBF) as the kernel function of SVM because the preliminary analysis showed that RBF performed superior in comparison with the other three kernel functions (linear, polynomial, and sigmoid). Here, the grid search approach was employed to optimize the two parameters of RBF-SVM: The penalty parameter *C* and the kernel parameter γ. These parameters were tuned with the following search space:

(1) $$\{\begin{array}{c} 2^{-5}\leq C\leq 2^{15}withstep\Delta C=2\\ 2^{-15}\leq \gamma \leq 2^{15}withstep\Delta \gamma =2^{-1} \end{array}$$

### 4.4. Ten-Fold Cross-Validation

Generally, three cross-validation (CV) methods, namely an independent data set test, a sub-sampling (or κ-fold CV) test, and a leave-one-out CV (LOOCV) test, are used to evaluate the anticipated success rate of a predictor [32,75]. In this study, we used a 10-fold CV to examine the proposed models. In the 10-fold CV, the benchmarking dataset was randomly partitioned into 10 subsets. One subset was used as a test set and the remaining nine subsets were used as the training sets. This procedure was repeated 10 times, with each subset being used once as a test set. The performance of the 10 corresponding results are averaged, with the outcome implying the performance of the classifier.

### 4.5. Feature Selection

Feature selection is one of the most important steps in developing ML-based models, and improve classification performance. In this study, we used the F-score algorithm along with a sequential forward search strategy to identify the optimal features [76]. Initially, the F-score algorithm was used to rank all the features and sort them from the highest to the lowest scores and thereby generate a ranked feature list. Later, features were added one by one from the ranked list, developing their corresponding predicting models. Finally, the feature subset that achieved the highest accuracy was regarded as the optimal features.

### 4.6. Performance Evaluation of ACPs Prediction

To evaluate the performance of the proposed method, the following four commonly used metrics [39,77,78,79,80,81,82] were employed: Sensitivity (SN), specificity (SP), accuracy (ACC), and the Matthews correlation coefficient (MCC), which are computed using the following formula:

(2) $$\{\begin{array}{c} \begin{array}{c} SN=\frac{TP}{TP+FN}\\ SP=\frac{TN}{TN+FP} \end{array}\\ \begin{array}{c} ACC=\frac{TP+FN}{TP+TN+FN+FP}\\ MCC=\frac{TP*TN-FP*FN}{\sqrt{\left(TP+FN\right)\left(TP+FP\right)\left(TN+FP\right)\left(TN+FN\right)}} \end{array} \end{array}$$

where TP and TN are the number of ACPs correctly predicted and non-ACPs correctly predicted, respectively. FN is the number of ACPs predicted as non-ACPs, whereas FP is the number of non-ACPs predicted as ACPs.

## Figures and Tables

**Table 1:** The best performance achieved by various feature encodings using optimal features.

Feature Encoding	Dimension	MCC	Accuracy	Sensitivity	Specificity
AAC	20	0.763	0.882	0.876	0.887
DPC	135	0.762	0.880	0.838	0.921
CTD	140	0.711	0.853	0.842	0.865
AAIF	143	0.775	0.887	0.872	0.902
QSO	99	0.734	0.867	0.846	0.887
CTF	133	0.698	0.844	0.929	0.759
NC5	54	0.706	0.852	0.808	0.880

**Table 2:** Performance of various methods on the independent dataset.

Methods	MCC	Accuracy	Sensitivity	Specificity	AUC	*p* Value
mACPpred	0.829	0.914	0.885	0.943	0.967	–
SVMACP [27]	0.592	0.768	0.554	0.981	0.896	0.000382
RFACP [27]	0.511	0.707	0.414	1.000	0.891	0.000401
iACP [30]	0.338	0.667	0.580	0.753	0.747	<0.00001

**Table 3:** Classification of 20 amino acids according to the seven specific types of physicochemical properties.

Properties	Class1	Class2	Class3
Hydrophobicity	Polar E, D, K, N, Q, R	Neutral A, G, H, P, S, T, Y	Hydrophobicity C, L, V, I, M, F, W
Normalized Van der Waals volume	0–2.78 A, C, D, G, P, S, T	2.95–4.0 E, I, L, N, V, Q	4.03–8.08 M, H, K, F, R, Y, W
Polarity	4.9–6.2 L, I, F, W, C, M, V, Y	8.0–9.2 A, G, P, S, T	10.4–13.0 H, Q, R, K, N, E, D
Polarizability	0–0.108 A, D, G, S, T	0.128–0.186 C, E, I, L, P, Q, V, N	0.219–0.409 K, M, H, F, R, Y, W
Charge	Positive K, R	Neutral A, N, C, Q, G, H, I, L, M, F, P, S, T, W, Y, V	Negative D, E
Secondary Structure	Helix A, E, H, K, L, M, Q, R	Strand V, I, Y, C, W, F, T	Coil D, G, N, P, S
Solvent Accessibility	Buried A, C, F, G, I, L, V, W	Exposed D, E, K, N, Q, R	Intermediate M, S, P, T, H, Y

## Supplementary Materials

Supplementary materials can be found at https://www.mdpi.com/1422-0067/20/8/1964/s1.
