# Supplementary material for: mACPpred: A Support Vector Machine-Based Meta-Predictor for Identification of Anticancer Peptides
Source: Int J Mol Sci. 2019 Apr 22;20(8):1964. doi: 10.3390/ijms20081964 (PMC6514805; doi:10.3390/ijms20081964)
Supplement: Supplementary file 1 [file ijms-20-01964-s001.pdf]

mACPpred: A Support Vector Machine-Based Meta-Predictor for Identification of Anticancer Peptides

Vinothini Boopathi [1], Sathiyamoorthy Subramaniyam [2,3], Adeel Malik [4,5], Gwang Lee [6,*] Balachandran Manavalan [6,*] and Deok-Chun Yang [1,*]

1 Graduate School of Biotechnology, College of life science, Kyung Hee University, Yongin-si, Gyeonggi-do 17104, Republic of Korea; vinothini9327@gmail.com (VB) and dcyang@khu.ac.kr (D-cY);
2 Research and Development Center, Insilicogen Inc., Yongin-si 16954, Gyeonggi-do, Republic of Korea; moorthy@insilicogen.com (SS);
3 Department of Biotechnology, Dr. N.G.P. Arts and Science College, Coimbatore, Tamil Nadu, India.
4 Perdana University Centre for Bioinformatics, Serdang, Malaysia
5 Current Address: Department of Microbiology and Molecular Biology, College of Bioscience and Biotechnology, Chungnam National University, Daejeon 34134, Republic of Korea; adeel@procarb.org (AM);
6 Department of Physiology, Ajou University School of Medicine, Suwon 443380, Republic of Korea; glee@ajou.ac.kr (GL) and bala@ajou.ac.kr (BM)

* Correspondence: glee@ajou.ac.kr (GL), bala@ajou.ac.kr (BM) and dcyang@khu.ac.kr (D-cY)

Figure S1. Performance comparison of the optimal feature-based models and the excluded feature-based models.

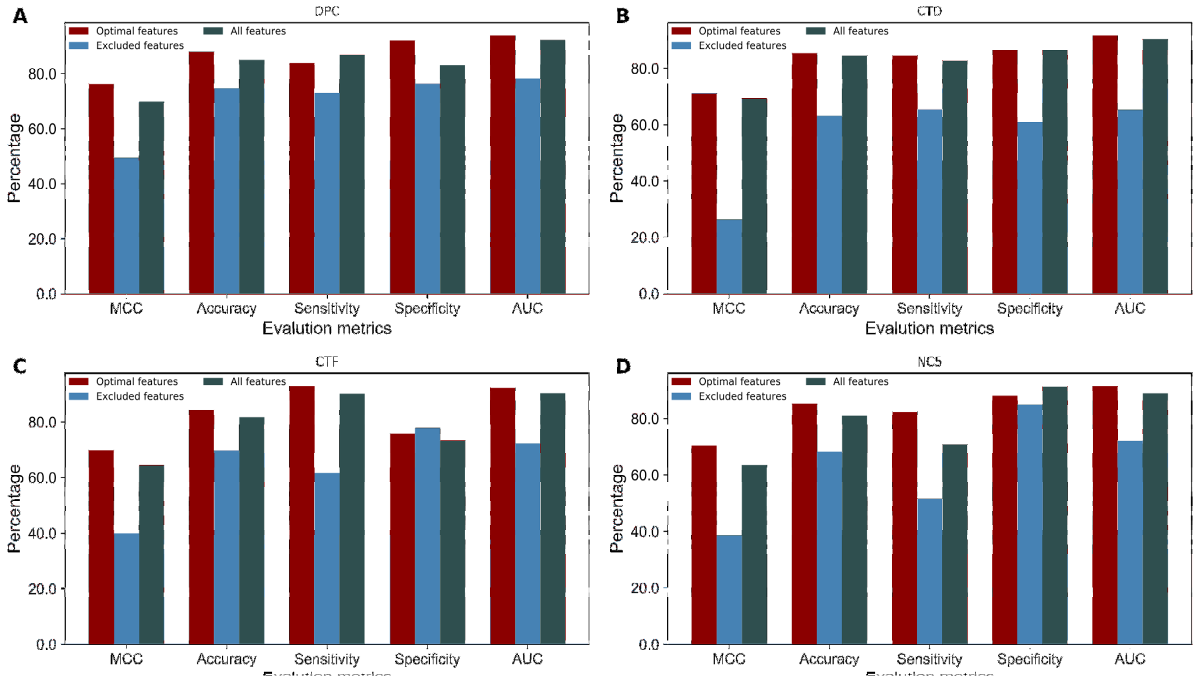

Table S1. Performance comparison of four different classifiers on seven different feature encodings.

Encodings	Classifier	MCC	Accuracy	Sensitivity	Specificity
AAC	SVM	0.7632	0.8816	0.8759	0.8872
	RF	0.7221	0.8609	0.8459	0.8759
	KNN	0.7307	0.8571	0.7519	0.9624
	LR	0.6880	0.8440	0.8383	0.8496
AAIF	SVM	0.7748	0.8872	0.8722	0.9023
	RF	0.6996	0.8496	0.8346	0.8647
	KNN	0.7225	0.8571	0.7820	0.9323
	LR	0.6880	0.8440	0.8421	0.8459
DPC	SVM	0.6997	0.8496	0.8684	0.8308
	RF	0.6922	0.8459	0.8647	0.8271
	KNN	0.6198	0.7970	0.6541	0.9398
	LR	0.6729	0.8346	0.7820	0.8872
CTD	SVM	0.6922	0.8459	0.8271	0.8647
	RF	0.6692	0.8346	0.8421	0.8271
	KNN	0.6457	0.8214	0.7744	0.8684
	LR	0.6432	0.8214	0.8383	0.8045
CTF	SVM	0.6446	0.8177	0.9023	0.7331
	RF	0.7231	0.8609	0.8910	0.8308
	KNN	0.3433	0.6447	0.9135	0.3759
	LR	0.6671	0.8327	0.8684	0.7970
QSO	SVM	0.7298	0.8647	0.8459	0.8835
	RF	0.6805	0.8402	0.8346	0.8459
	KNN	0.6361	0.8177	0.7932	0.8421
	LR	0.6808	0.8402	0.8233	0.8571
NC5	SVM	0.6340	0.8102	0.7068	0.9135
	RF	0.5873	0.7932	0.7669	0.8195
	KNN	0.5102	0.7538	0.8045	0.7030
	LR	0.6095	0.8045	0.7857	0.8233

Table S2. List of optimal features for each feature encoding obtained through two-step feature selection protocol.

Feature encodings	Selected features
DPC	F8, F9, F11, F13, F15, F17, F30, F31, F35, F36, F47, F49, F52, F57, F58, F64, F67, F72, F73, F75, F78, F79, F81, F89, F90, F100, F101, F102, F103, F104, F106, F107, F108, F110, F113, F120, F123, F126, F127, F129, F130, F143, F144, F145, F146, F148, F149, F150, F151, F152, F153, F155, F158, F160, F161, F162, F164, F171, F173, F176, F177, F181, F182, F185, F186, F187, F190, F191, F193, F197, F198, F199, F201, F202, F203, F207, F208, F209, F210, F211, F212, F214, F217, F221, F226, F228, F229, F232, F235, F236, F239, F242, F246, F248, F251, F262, F263, F276, F278, F282, F284, F289, F293, F294, F302, F305, F314, F316, F317, F318, F324, F343, F345, F346, F348, F349, F351, F353, F354, F357, F358, F361, F365, F368, F369, F376, F377, F378, F382, F383, F388, F390, F391, F392, F397
CTD	F1, F2, F3, F4, F5, F6, F7, F9, F10, F11, F12, F13, F14, F15, F16, F17, F18, F19, F20, F21, F22, F23, F24, F27, F28, F29, F30, F31, F32, F33, F34, F35, F36, F37, F38, F39, F40, F41, F42, F43, F44, F45, F46, F47, F48, F49, F50, F51, F52, F53, F54, F55, F56, F57, F58, F59, F60, F61, F62, F63, F64, F65, F66, F67, F68, F69, F70, F71, F72, F73, F74, F75, F76, F77, F78, F79, F80, F81, F82, F83, F84, F85, F86, F87, F88, F89, F90, F91, F92, F93, F94, F95, F96, F97, F98, F99, F100, F101, F102, F103, F104, F105, F106, F107, F108, F109, F110, F111, F112, F113, F114, F115, F116, F117, F118, F119, F120, F121, F122, F124, F125, F126, F127, F128, F129, F130, F131, F132, F133, F134, F136, F137, F138, F139, F141, F142, F143, F145, F146, F147
QSO	F1, F2, F3, F4, F5, F6, F7, F8, F9, F10, F11, F12, F13, F14, F15, F16, F17, F18, F19, F20, F21, F22, F23, F24, F25, F26, F28, F29, F30, F31, F32, F33, F34, F35, F36, F37, F38, F39, F40, F41, F42, F43, F44, F45, F46, F47, F48, F49, F50, F51, F52, F53, F54, F55, F56, F57, F58, F59, F60, F61, F62, F63, F64, F65, F66, F67, F68, F69, F70, F71, F72, F73, F74, F75, F76, F77, F78, F79, F80, F81, F82, F83, F84, F85, F86, F87, F88, F89, F90, F91, F92, F93, F94, F95, F96, F97, F98, F99, F100
AAIF	F1, F2, F3, F4, F5, F6, F7, F8, F9, F10, F11, F12, F13, F14, F15, F17, F18, F19, F20, F21, F22, F23, F24, F25, F26, F27, F28, F29, F30, F31, F32, F33, F34, F35, F36, F39, F40, F41, F42, F43, F44, F45, F46, F47, F49, F50, F51, F52, F53, F54, F55, F56, F65, F66, F67, F69, F70, F71, F72, F73, F74, F75, F76, F77, F78, F79, F80, F81, F82, F83, F84, F85, F86, F87, F88, F89, F90, F91, F92, F93, F94, F95, F96, F97, F98, F99, F100, F101, F102, F103, F104, F106, F108, F109, F110, F111, F112, F113, F114, F115, F116, F117, F118, F119, F120, F121, F122, F123, F124, F125, F126, F128, F129, F130, F131, F132, F133, F134, F135, F136, F137, F139, F140, F141, F142, F143, F144, F145, F146, F147, F148, F149, F150, F151, F152, F153, F154, F155, F156, F157, F158, F159, F160

NC5	F3, F4, F5, F6, F9, F12, F14, F23, F24, F30, F32, F41, F42, F43, F44, F50, F58, F59, F64, F69, F71, F75, F82, F83, F85, F89, F92, F94, F99, F100, F103, F104, F109, F118, F119, F122, F124, F129, F138, F139, F143, F144, F149, F163, F165, F169, F172, F182, F183, F188, F189, F190, F197, F198
CTF	F11, F14, F15, F16, F17, F18, F33, F35, F38, F39, F41, F46, F57, F60, F61, F62, F64, F65, F69, F72, F73, F74, F75, F76, F79, F80, F100, F102, F105, F106, F108, F112, F113, F114, F119, F120, F122, F123, F124, F126, F127, F129, F132, F133, F134, F137, F139, F160, F162, F173, F175, F178, F179, F182, F183, F184, F185, F186, F187, F188, F189, F190, F192, F193, F194, F195, F201, F208, F216, F217, F219, F228, F229, F231, F232, F234, F245, F246, F247, F248, F249, F250, F259, F264, F266, F270, F274, F275, F276, F281, F293, F295, F296, F297, F298, F300, F319, F321, F326, F328, F339, F340, F341, F342, F343
